# Molecular genetic and clinical characteristic analysis of primary signet ring cell carcinoma of urinary bladder identified by a novel OR2L5 mutation

**DOI:** 10.1002/cam4.5121

**Published:** 2022-08-11

**Authors:** Mohammed Alradhi, Shuang Wen, Mohammed Safi, Abdullah Al‐danakh, Honglong Wang, Abdullah Shopit, Min Sun, Bo Fan, Xiancheng Li

**Affiliations:** ^1^ Department of Urology, Second Affiliated Hospital of Dalian Medical University Dalian China; ^2^ Department of Pathology, Dalian Friendship Hospital Dalian China; ^3^ Department of Respiratory Diseases Shandong Second Provincial General Hospital Shandong University Shandong China; ^4^ Department of Urology, First Affiliated Hospital of Dalian Medical University Dalian China; ^5^ Department of Pharmacology, Dalian Medical University Dalian China; ^6^ Department of General Surgery, Taihe Hospital, Hubei University of Medicine Hubei China

**Keywords:** immunohistochemistry, OR2L5, primary signet ring cell carcinoma, urinary bladder, whole‐exome sequencing

## Abstract

To get a better understanding of the genetic basis of primary signet ring cell carcinoma (SRCC) of the bladder, which is highly rare and not yet explored. First, by using immunohistochemistry to find histological pathological characteristics. Second, a massively parallel whole‐exome sequencing (WES) was performed on a 58‐year‐old male patient who had painless macroscopic hematuria and was pathologically diagnosed with primary SRCC of the bladder, followed by comparing with genes of ordinary urothelial cancer (UC) from TCGA. Furthermore, a population‐based analysis using the SEER database was performed to investigate the prognosis (SRCC vs. UC). We identified 63 copy number variations (CNVs) with gain counts and 181 CNVs with loss counts. Totally 4515 mutations were discovered in C > T with a success rate of greater than 89%. The most frequently mutated pathway was RTK‐RAS which has 85 genes involved in carcinogenic signaling. Final screening on predisposing genes is performed after filtering based on ACMG. Moreover, several driver genes, including NBN, KCTD18, SPATA13, ANKRD36, OR2L5, MALRD1, and LSMEM1, were detected. Sanger sequencing of germline DNA revealed the presence of a mutant base A/G of OR2L5 in the sequence, which was discovered for the first time in primary SRCC of the bladder. Furthermore, the immunohistochemical profile showed that primary SRCC of the bladder were positive for CK7, CK20, GATA‐3, and expression of CK(AE1/AE2), EMA, and Ki67. In the SEER‐based study, the patients with primary SRCC of the bladder got a worse prognosis compared to those with UC with median months overall survival (OS) 14 vs. 41, respectively, *P* = 0001, even after adjusting the variables in the Cox regression model, the SRCC of the bladder showed worse survival HR = 1.119, 95% CI = (1.081–1.328), *P* = 0.0001. These results imply that suppression of potential driver mutations may be a viable adjuvant treatment approach for primary SRCC in the bladder in place of standard chemotherapy, a possibility that warrants further clinical investigation.

## Graphical abstract

Detailed methodology diagram of signet ring cell carcinoma of bladder.

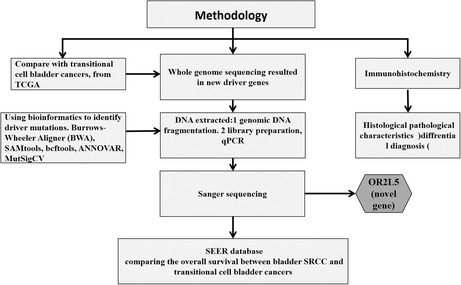

## 1 INTRODUCTION

Primary signet ring cell carcinoma (SRCC) is a rare bladder adenocarcinoma. Histologically, SRCC is classified under mucinous adenocarcinoma and represents 0.24%–2% of primary epithelial urinary bladder cancers.^1^, ^2^ According to the World Health Organization, these are tumors in which more than 50% of the tumor's pathophysiology is composed of mucin located extracellularly and malignant epithelial cells that form individual cells, clusters, or layers.^3^, ^4^ Mucosal epithelial cells secrete mucin, which is a high molecular weight glycoprotein consisting of oligosaccharides linked to a core protein as a lubricant to protect themselves from infections and irritants.^5^ So far, the histopathogenesis of primary mucin‐producing adenocarcinomas, such as SRCC, is unclear due to the absence of columnar or mucus‐secreting glandular epithelium in the normal bladder.^6^ Two different patterns of urothelial metaplastic potential were suggested by researchers. The first developed as a result of hyperplastic epithelial buds invading the lamina propria (Von Brunn's nest) and is called Cystitis cystica, while the second pattern is Cystitis glandular, which is a lesion considered premalignant, caused when the covering layer of these cysts metaplasia urothelial to columnar mucin‐producing cells.^7^ Moreover, in the absence of downward invagination with chronic vesical irritation and infection functioning as risk factors, cuboidal or columnar metaplasia of the surface epithelium can occur.^8^ Mucin deposition can occur both extracellularly and intracellularly, resulting in the creation of a distinctive signet ring; despite the majority of these tumors releasing mucin, mucus passing during micturition is uncommon.^9^ A gene set enrichment analysis (GSEA) revealed that the EMT and “StemCell Up‐Regulation” pathways were enriched for SRCC‐specific, upregulated genes. Furthermore, scientists have proposed ideas on the transmission of advanced disease pathology based on the discovery that SRCCs demonstrate biological activity in gastric cancer.^10^ The two most prominent pathogenic hallmarks of SRCCs are mucin accumulation which contributes to their widespread pattern of dissemination and aberrant cell–cell adhesion.^11^ The genetic and molecular features of primary SRCC of the urinary bladder have not been studied yet. Mutations, predisposing genes, driving genes, and pathways all have a role in the development of bladder SRCC, making this a crucial area of study. We report the results of unbiased sequencing of all protein‐coding exons and compare them to patient‐matched normal controls, which led to the discovery of mutations that provide light on the tumor's biology. Next‐generation sequencing (NGS) offers convincing evidence to detect cancers' genomic changes. Single nucleotide polymorphisms (SNPs), INDEs, somatic single nucleotide variations (SNVs), insertion and deletions (INDELs), copy number variations (CNVs), predisposing genes, and driver genes have been identified between primary bladder SRCC and surrounding normal tissue specimens.

## 2 MATERIALS AND METHODS

### 2.1 Analysis of histological and pathological features

#### 2.1.1 Patients data extraction

**FIGURE 1:**
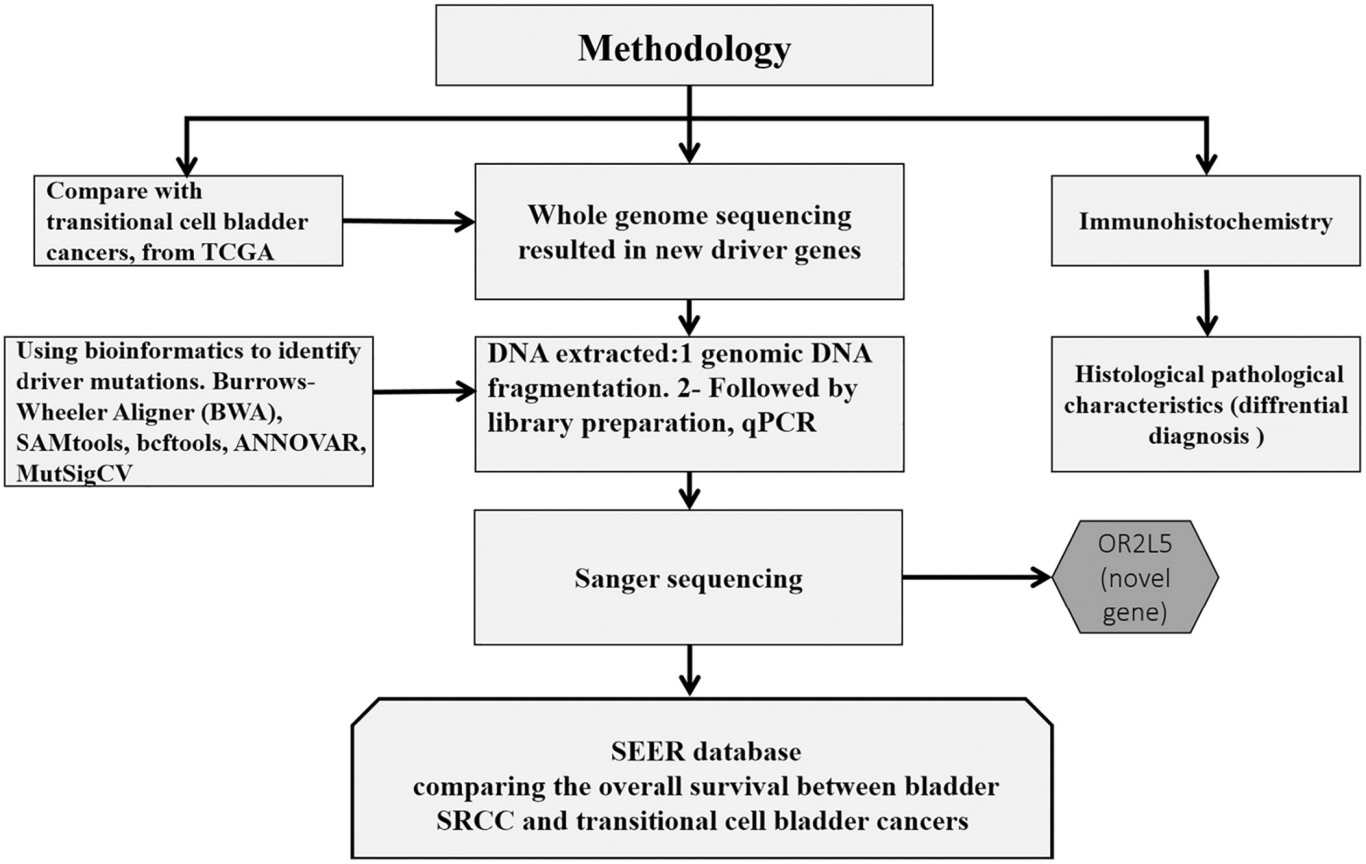
A flowchart summarizes the mean methodology parts completed throughout the research.

The clinical findings data, the sources, and information on the experimental and the control tissues were obtained from the Second Hospital of Dalian Medical University. The study protocol was approved by the Ethics Committee of the Second Hospital of Dalian Medical University and followed the Declaration of Helsinki Ethical Principles for Medical Research Involving Human Subjects. The patient's written consent was taken in accordance with Dalian's ethics committee for human research. To organize and define all of the mean methodology parts completed throughout the research, a flowchart is constructed (Figure 1).

#### 2.1.2 Immunohistochemistry

A multiple transurethral resection of bladder tumor chips measuring 3 ml in volume was received in the laboratory of our hospital. The samples were fixed in 10% formaldehyde, embedded in 4 mm paraffin, and stained with hematoxylin and eosin (HE) before being put on slides and photographed under a light microscope. Immunohistochemistry was performed on tissue slices that had been formaldehyde‐fixed and paraffin‐embedded (FFPE), with normal bladder tissues used as a positive control. At 4°C for 24 h, the slices were treated with various antibodies such as CK(AE1/AE2) (mouse monoclonal antipan cytokeratin (AE1/AE3) (dilution 1:200, A500‐019A, Invitrogen), EMA (Mouse Monoclonal anti‐EMA/MUC1) (dilution 1:200; MA5‐11202; Invitrogen); CK7 (rabbit monoclonal anti‐Cytokeratin 7) (dilution 1:1000, ab 68459, Abcam), CK20 (rabbit monoclonal anti‐Cytokeratin 20) (dilution 1:200, ab76126, Abcam), GATA‐3 (rabbit monoclonal anti‐GATA3) (dilution 1:500, ab 199428, Abcam), Ki67 (rabbit polyclonal anti‐Ki67) (dilution 1:6000, 27,309‐1‐AP, ProteinTech Group), CDX‐2 (rabbit monoclonal anti‐CDX2) (dilution 1:1000, ab76541, Abcam), villin (rabbit monoclonal anti‐Villin) (dilution 1:100, ab130751, Abcam), and β‐catinin (rabbit monoclonal anti‐beta Catenin) (dilution 1:500, ab130751, Abcam), ER (rabbit monoclonal antiestrogen Receptor alpha) (dilution 1:200, ab108398, Abcam), PR (rabbit monoclonal antiprogesterone Receptor) (dilution 1:400, ab16661, Abcam); PSA (rabbit polyclonal anti‐PSA) (dilution 10 μg/ml, PA5‐119384, Invitrogen), and PSAP (rabbit polyclonal anti‐PSAP) (dilution 1:400, PA5‐82153, Invitrogen).

#### 2.1.3 HE and AB‐PAS staining

We examined the tissues by the HE and AB‐PAS and rinsed them in PBS and fixed with Carnoy's fixative (Solarbio, Beijing, China) at 4°C. The tissues were dehydrated and cleared before being dipped in wax and sliced into 5‐m‐thick slices. Afterward, the slices were dewaxed and stained with an HE or AB‐PAS Stain Kit (Solarbio, Beijing, China).

### 2.2 Whole‐genome sequencing

#### 2.2.1 Quality control and insert length analysis

In raw sequencing data, there are a few adaptor reads, along with low‐quality nucleotides and previously unexplained nucleotides. To assure the accuracy of the analysis, raw readings must be filtered to obtain clean reads, and subsequent analyses will be performed with clean reads. Paired reads polluted by adapters, reads with more than 10% unknown bases, and reads with more than 50% low‐quality (Phred grade 5) bases were removed. High‐quality clean data were acquired and utilized to guarantee that a full downstream analysis could be conducted. We perform statistics on raw data quality values and other information and use FastQC to visually evaluate the quality of the sequencing data of the samples. Examining whether insert length distribution is in line with expectations can reflect the library preparation experiment's quality. We used the Read1 and Read2, obtained by pair‐end sequencing (Pair‐End) containing three very useful relationship information: connection to each other, distance, and sequence direction. This information is the key signal for genomic variation detection, especially structural variation detection.

#### 2.2.2 Extraction of the DNA

The genomic DNA from a primary SRCC of the bladder sample, as well as any adjacent or surrounding normal tissue, were extracted using a kit from GeneRead DNA FFPE following the manufacturer's protocol (Qiagen, Germany). Two methods were used to verify the integrity of isolated genomic DNA: a Qubit® 2.0 Fluorometer (Invitrogen, USA) and Qubit® DNA Assay kit, and electrophoresis with 1% agarose gel for monitoring DNA fragmentation and contamination.

#### 2.2.3 Library sample steps of preparation and sequencing

Using covaris technology, genomic DNA fragmentation was performed at the 180–280 bp level. We got the WES using the Illumina HiSeq X platform at the (Biologics DNA sequencing center, Shanghai, China) by using an Agilent SureSelect Human All Exon Kit following the manufacturer's instructions (Agilent Technologies, CA, USA). The products were processed and measured using the Beckman Coulter AMPure XP system, which was purchased from Beckman Coulter (Beverly, USA), and a high‐sensitivity DNA test was performed using the Agilent Bioanalyzer 2100 system. DNA libraries with an estimated 150 bp insert size were read using an Illumina Hi‐Resolution Sequencing System at the Novogene sequencing facility (Illumina, San Diego, CA, USA).

#### 2.2.4 Using bioinformatics

With the Burrows‐Wheeler Aligner (BWA) software, valid sequencing reads were aligned to the reference genome (UCSC hg19 and hg 38), leading to generating BAM files. After sorting the BAM files with SAMtools, Picard (http://broadinstitute.github.io/picard/) was used to identify duplicate readings. We used Picard to identify these duplicates for further research. Finally, the sequence coverage and depth were calculated using the final file of BAM.

#### 2.2.5 Variant calling, indel detection, and somatic mutation detection

To ensure a relevant study, we revealed and sorted out variants (SNPs and INDELs) using bcftools and mpileup in the SAMtools. Variant call forms were described and annotated using ANNOVAR based on the 1000 Genomes databases and dbSNP as well as more comparable databases already in existence. The Genome Analysis Toolkit (gatk) offers the most effective detection method for mutect2, which is separated into three modes as follows: (I) tumor‐normal mode (tumor sample and paired normal control sample), (II) comparative data for a particular tumor pattern, (III) mitochondrial pattern. The muTect software is mostly used to identify somatic SNV‐InDel sites. MuTect was used to detect somatic SNVs, while Strelka was used to detect somatic INDELs. To identify changes in somatic CNVs, control‐FREEC was used.

#### 2.2.6 Kataegis‐rainful analysis

The precipitation Moritz Goretzky claimed that Plot could depict the hypermutated genomic area (Kataegis loci) by charting the mutation pitch at the chromosomal scale. Using Kategis' criterion, we found hypermutated genomic sites (six consecutive mutations per 1000 bp distance on average). Beginning with the baseline site sequence, add six subsequent mutations and calculate the average mutation distance. If the average interchange distance exceeds 1000, one element is added to the back of the queue, and one is removed from the front. If the average mutation distance is less than or equal to 1000, increase the mutation until it is more than 1000. Following that, all of the sequence's mutations are recorded and generated as a kataegis.

#### 2.2.7 Identification of predisposing genes

Predisposing genes are genes that can encode genetic diseases or obtain disease susceptibility under appropriate environmental stimuli. We filter the SNP/InDel detected above to obtain the mutation sites that may cause disease. Then compare the mutation‐related CGC database to identify potential disease susceptibility genes. The specific filtering method for mutation sites is to (1) filter out variant sites with a depth of less than 10x; (2) filter the mutation sites in the 1000 Genome Database (the frequency in the population is greater than 0.01), remove the diversity sites between individuals, and get the rare mutations that may cause disease (rare); (3) keep the exonic region (exonic) or splicing site (splicing, 2 bp upstream of the splicing site) mutations, that is, filter out the mutations located in the intergenic region, noncoding region, and intron region point; (4) remove synonymous mutations (mutations that do not cause amino acid coding changes) to obtain mutations that affect gene expression products (reserved mutations include frameshift and nonframeshift mutations in InDel); and to (5) filter according to the scores of SIFT, polyphen2_hvar, and polyphen2_hdiv and at least evaluate as harmful mutations in one database or evaluate as moderately harmful mutations in two or more databases. SNP harmfulness prediction, the dbNSFP database is an annotated database for nonsynonymous mutations. It mainly evaluates the conservation and pathogenicity of amino acids through corresponding calculated scores. These scores include SIFT scores, PolyPhen2, HDIV, scores, PolyPhen2 HVAR, scores, LRT scores, MutationTaster, scores, MutationAssessor score, FATHMM scores, GERP++ scores, PhyloP scores, and SiPhy scores. This research is mainly screened based on the mutation classification predicted by SIFT (score ≤ 0.05) and PolyPhen2_HVAR (score ≥ 0.909). The following is how the score might be interpreted: 0.00–0.05—variants with scores in this range are thought to be harmful, whereas a variant with a PolyPhen score of 0.0 is predicted to be benign. In summary, SAM tools software was used to investigate germline mutations (SNVs and INDELs) in normal tissues from patients. Potential predisposing genes were found by identifying germline mutations and comparing them to the CGC (http://cancer/sanger.ac.uk/cancergenome/projects/).

#### 2.2.8 Identification of potential driver mutations

High‐frequency mutation genes can be detected from a large number of samples, and these genes can be used as candidate genes for the occurrence and development of disease. After finding the driver genes, patients can be targeted for personalized precision medical treatment. We use the SNP and InDel information obtained from the previous analysis of all samples to extract potential driver genes through the driver gene analysis software Oncodrive CLUSTL (an updated version of Oncodrive CLUST). At the same time, we also use MutSigCV to obtain high‐frequency mutation genes and use various software to accurately identify driver mutations. Finally, we compare our resulting driver genes with differentially expressed genes of ordinary transitional cell cancer of the bladder from the TCGA database.

### 2.3 Population‐based study from SEER‐database

#### 2.3.1 Data resource

The ethics statement permission to access the SEER research data files was obtained by using the reference number 10237‐Nov2019. The data released by the SEER database do not require informed patient consent, with additional treatment fields through SEER* Stat software (version 8.4.0.1), released in May 2022, and represent the largest collection of the primary SRCC. We extracted patients with the histology SRCC (ICD‐O‐38490/3) or UC. UC included ICD‐O‐38120/3 (transitional cell carcinoma), ICD‐O‐38122/3 (transitional cell carcinoma, spindle cell), and ICD‐O‐38131/3 (transitional cell carcinoma, micropapillary), The diagnosis was confirmed by positive histology. The following variable information were selected as the prognostic factors in our study: age at diagnosis, race, sex, primary site, marital status, grade, SEER historic stage, type of surgery, the reason for noncancer‐directed surgery, radiation, the sequence of radiation with surgery, information on chemotherapy treatment, SEER classification of other causes of death, lymph node removal, insurance status, marital status, and vital status record. The study period was calculated till the last update of the SEER database. The age of diagnosis was grouped into 20–69 years and ≥70 years, and marital status was divided into married, unmarried, and others. The race was divided into White, Black, and others. The reason for noncancer‐directed surgery was used as an indicator of surgical history, which we grouped into surgery performed and surgery not performed (not recommended, not performed, and unknown). Type of surgery is grouped into (no surgery, TURBT, partial cystectomy, radical cystectomy, pelvic exenteration, and other types of surgery). Radiation is also grouped into radiation (beam radiation, implants, radiation not otherwise specified), no radiation, or other. Information on chemotherapy treatment and the cause of death by the same cancer or other causes were also obtained.

#### 2.3.2 Statistical analysis

The demographic and tumor characteristics of patients in both groups were compared using χ2. OS based on the primary site was estimated by KM, and comparisons among groups were carried out by a log‐rank test. Univariate and multivariate Cox proportional hazards models were used to determine the effect of a prognostic variable on OS. SPSS version 26.0 (SPSS Inc., Chicago, IL, USA) was used to determine statistical significance at *P* < 0.05 and limits of 0.0001.

## 3 RESULTS

### 3.1 Analysis of histological and pathological features

#### 3.1.1 Case presentation

A 58‐year‐old man presented to our hospital with painless macroscopic hematuria; the urine routine examination revealed positive occult blood and no evident abnormalities in PSA, CA199, CA724, or CEA; he then had TURBT. The pathology report revealed that the patient had high‐level invasive urothelial cancer. The majority of the bladder's base layer is glandular. The macroscopic examination revealed a 7*7*4 cm tumor and a 1.3*0.5*0.5 cm ulcer in the near triangular area of the left wall of the bladder, reaching as deep as the surrounding fat (Figure S1B). The remaining bladder mucosa was edematous. Primary SRCC in bladder glandular cystitis was the pathological diagnosis, a poorly differentiated carcinoma with more than 50% signet ring cells. Tumor cells were organized into lobules that were divided by fibrovascular septae. There were 1–2 mitoses/hpf and areas of tumor necrosis. Robot‐assisted laparoscopic radical cystectomy + ileal conduit + pelvic lymph node dissection were done (Figure S1A).

#### 3.1.2 Immunohistochemical profile

**FIGURE 2:**
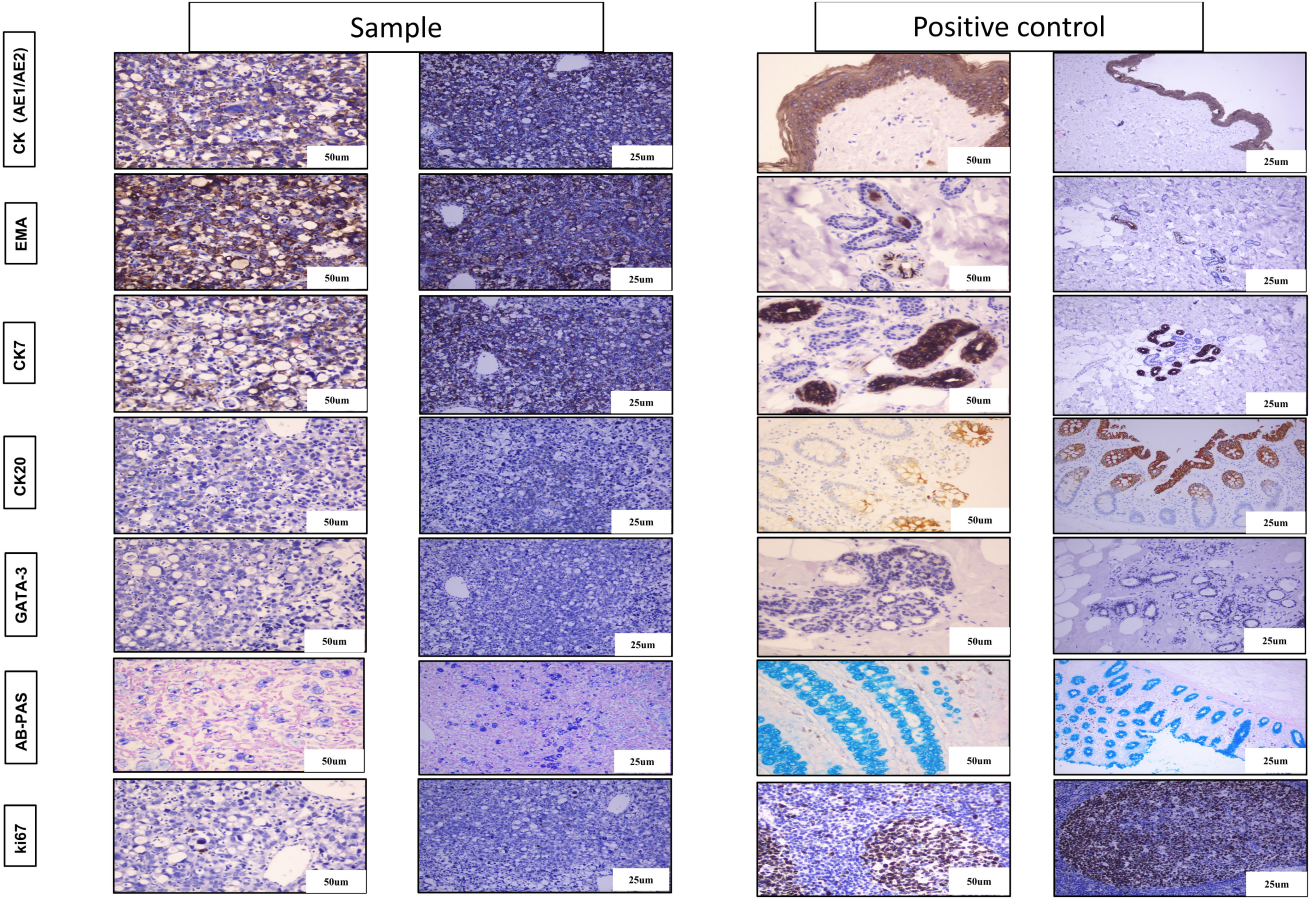
Images of the tumor cells from the primary signet ring cell carcinoma (SRCC) of the bladder (scale bar: 50 mm, 25 mm). The figures representative IHC images for CK7, CK20, and GATA‐3 which are the marker of the primary SRCC of the bladder, Ki‐67 proliferation index was 20%–30%, AB‐PAS showed a low expression level. In addition, CK(AE1/AE2), EMA is the marker of epithelial tissue origin.

**FIGURE 3:**
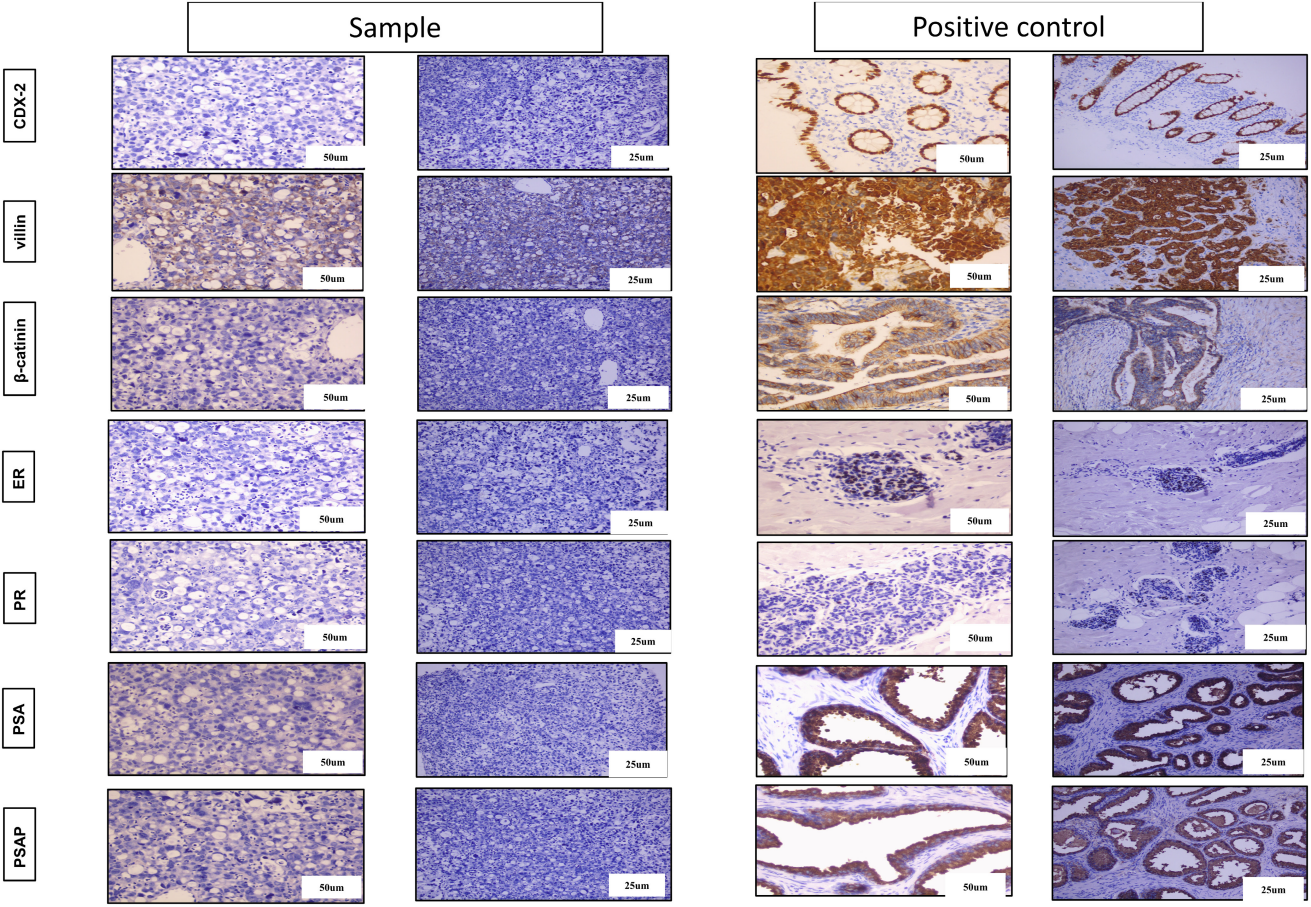
IHC images of the indicators for differential diagnosis of primary bladder SRCC such as secondary SRCC and metastasis tumors. Representative IHC images for CDX‐2, villin, and β‐catinin were the markers for excluding gastrointestinal origin, ER (estrogen receptor), PR (progesterone receptor) (ER and PR were the markers for exclusion of breast cancer origin), PSA, PSAP (were the markers for exclusion of prostate origin).

Immunohistochemical analysis revealed that CK7, CK20, and GATA‐3 were all overexpressed (+++), indicating that the patient had primary SRCC of the bladder. The Ki‐67 proliferation index was positive expression (20%–30%), which may be associated with prognosis, and low AB‐PAS expression level (Figure 2). In addition, CK(AE1/AE2), EMA, CDX‐2, villin, β‐catinin, PSA, PSAP, ER, and PR both showed moderately express, which indicates the epithelial origin of the tissue (Figure 3). differential diagnosis of metastatic SRCC from secondary or metastatic SRCC; CDX‐2 (with no expression), villin, and β‐catinin (with low expression) exclude the gastrointestinal origin of the tumor (Figure 3). Moreover, low expression of PSA, PSAP, and ER, PR were the markers for exclusion of prostate origin, and breast cancer origin, respectively (Figure 3).

### 3.2 Whole‐genome sequencing

#### 3.2.1 Sequencing data evaluation, quality control, and insert length analysis

**FIGURE 4:**
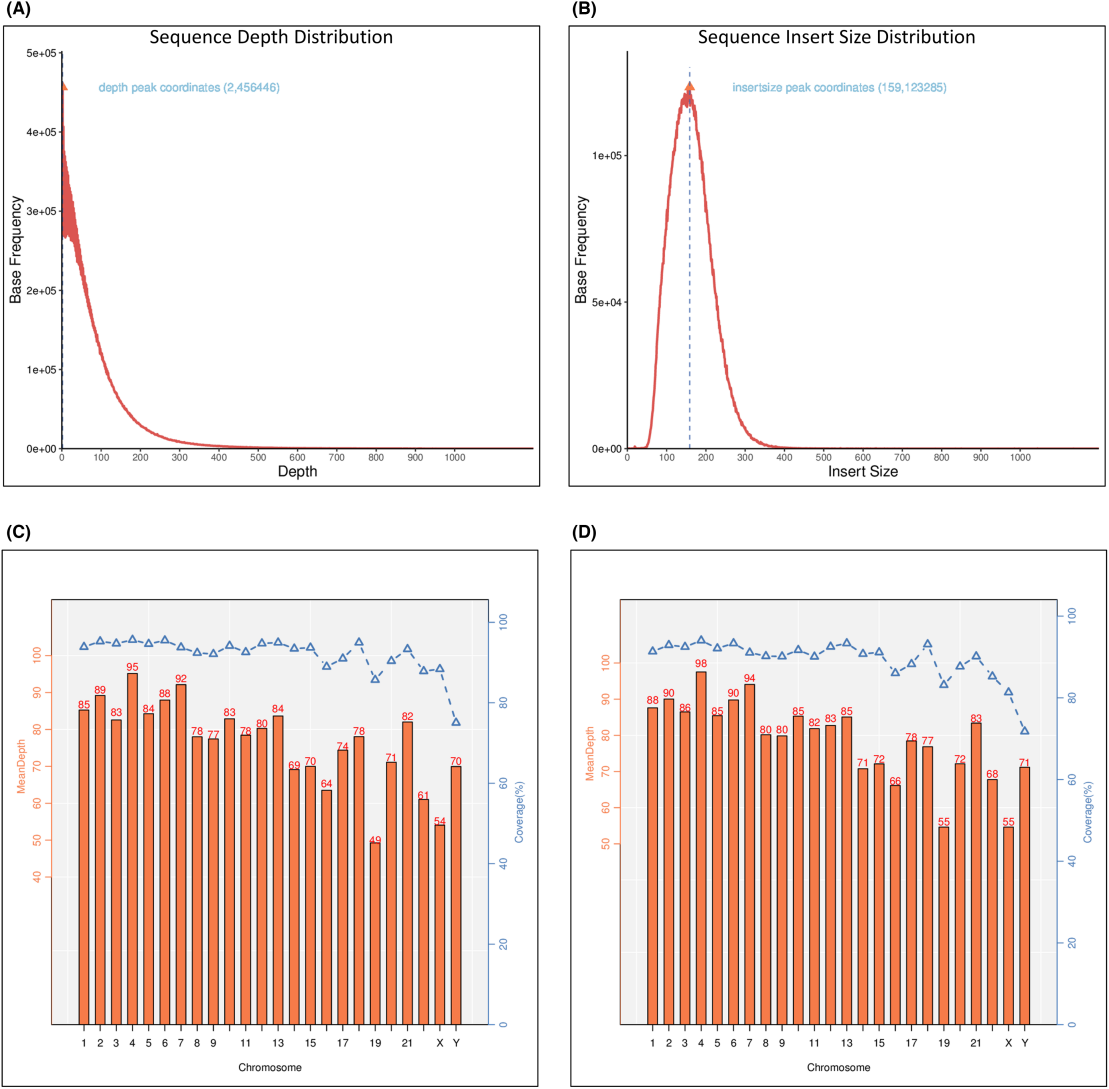
The sequence distribution. (A) Sample insertion fragment length distribution density plot the horizontal axis is the length of the inserted fragment, and the vertical axis is the statistical number corresponding to the length of an inserted fragment. (B) Sample sequencing depth density plot of the horizontal axis is the sequencing depth, and the longitudinal axis is the statistical number corresponding to the sequencing depth of a certain base (C) for tumor sample and (D) for the normal sample average coverage depth column chart and coverage line chart A. The abscissa represents the chromosome number, the left ordinate indicates the average depth of coverage, and the right ordinate represents coverage. Depth of coverage per chromosome = amount of sequencing data per chromosome/total length of exon regions on each chromosome. Coverage = total length of each chromosome covered/total length of exon regions on each chromosome.

We perform statistics on raw data quality values and other information and use FastQC to visually evaluate the quality of the sequencing data of the sample. The total reads account was 44,023,636 vs. 44,655,148 in both tumor and normal tissues, respectively. The overall average of Q30 was more than 90%, whereas the error rate was less than 0.1%. The original data must be filtered to obtain clean data (Table 1). Insert size of the length distribution with Read1 and Read2 obtained by pair‐end sequencing contains three very useful relational information: interconnected, distance, and sequence direction (Figure 4A). Sample sequencing depth density map may show the accuracy of variation detection. The deeper the sequencing coverage of challenging information, the more regions covered in the reference genome, the more mutation sites may be found, and the more information represented in the sequencing data. The average exon/capture region sequencing depth is not statistically significant. This information is a key signal for genomic variation detection, especially structural variation detection (Figure 4B). The depth column of coverage per chromosome = amount of sequencing data per chromosome/total length of exon regions on each chromosome. Coverage = total length of each chromosome covered/total length of exon regions on each chromosome, the tissue of tumor, and normal Sample average coverage depth column chart and coverage line chart (Figure 4C,D).

**TABLE 1:** Statistics of the original data of each sample

	Raw data tumor tissue (SRCC bladder)	Raw data normal tissue	Clean data Tumor tissue (SRCC bladder)	Clean data normal tissue
Total reads count (#)	44,023,636	44,655,148	43,452,308	43,915,652
Total bases count (bp)	6,603,545,400	6,698,272,200	6,394,784,628	6,446,601,595
Average read length (bp)	150	150	147	147
Q20 bases count (bp)	6,378,490,255	6,463,035,949	6,201,376,493	6,249,097,285
Q20 bases ratio (%)	96.59%	96.49%	96.98%	96.94%
Q30 bases count (bp)	6,037,602,999	6,113,859,619	5,878,623,584	5,921,837,718
Q30 bases ratio (%)	91.43%	91.28%	91.93%	91.86%

*Note*: The overall average of Q30 was more than 90%, whereas the error rate was less than 0.1%. The original data must be filtered to obtain clean data.

#### 3.2.2 Identification of SNPs and INDELs

A people's genome typically has 3.6 million SNPs. The dbSNP is a cataloged database of high‐frequency SNPs. Further highlights the number of SNPs and INDEL discovered in various parts of the genome and coding regions in the primary bladder SRCC and surrounding specimens (Table 2). They were mainly distributed in exonic, intronic, intergenic, and other locations. In addition, each individual has a 350 K INDEL that was discovered across the genome. Table 2 also illustrates the number of distinct INDEL and SNP types found in the genomic and coding sections of the human genome. In primary bladder SRCC and normal samples, a total of 92,280 and 84,089 SNPs were discovered and were mostly present in the coding sequence (CDS) regions, intronic, and intergenic. On the other hand, the INDEL quantity in both primary SRCC and normal tissue was 962,988, respectively, in the genome and coding regions (Table 2). The primary bladder SRCC and surrounding specimens were mainly distributed in exonic, intronic, intergenic, and other locations. In addition, each individual has a 350 K INDEL that was discovered across the genome. Table 2 shows the number of distinct INDEL types found in the genomic and coding sections of the human genome.

**TABLE 2:** The number of SNPs and INDELs in different regions of the genome and in coding regions

	SNP			INDELs	
Sample	Primary tumor	Normal	Sample	Primary tumor	Normal
CDS	78,151	70,153	CDS	723	761
Synonymous_SNP	28,657	25,343	frameshift deletion	272	263
Missense_SNP	45,366	41,030	frameshift insertion	209	244
Startloss	93	74	nonframeshift deletion	129	119
Stopgain	3028	2698	nonframeshift insertion	72	76
Stoploss	15	10	Startloss	2	0
Unknown	299	261	Stopgain	42	49
Intronic	15	12	Stoploss	1	3
UTR3	3	6	Unknown	3	6
UTR5	30	26	Intronic	0	0
Splicing	28	30	UTR3	0	0
ncRNA_exonic	178	145	UTR5	1	2
ncRNA_intronic	11	3	Splicing	9	1
ncRNA_UTR3	0	0	ncRNA_exonic	3	2
ncRNA_UTR5	0	0	ncRNA_intronic	0	0
ncRNA_splicing	0	0	ncRNA_UTR3	0	0
Upstream	23	21	ncRNA_UTR5	0	0
Downstream	1	772	ncRNA_splicing	0	0
Intergenic	20	23	Upstream	0	0
frameshift deletion	‐	‐	Downstream	0	0
frameshift insertion	‐	‐	Intergenic	2	2
Others	279	249	Others	12	8
Total	78,473	70,438	Total	738	769

#### 3.2.3 Identification of SNPs/INDELs mutations, CNV mutation, and SV mutation

**FIGURE 5:**
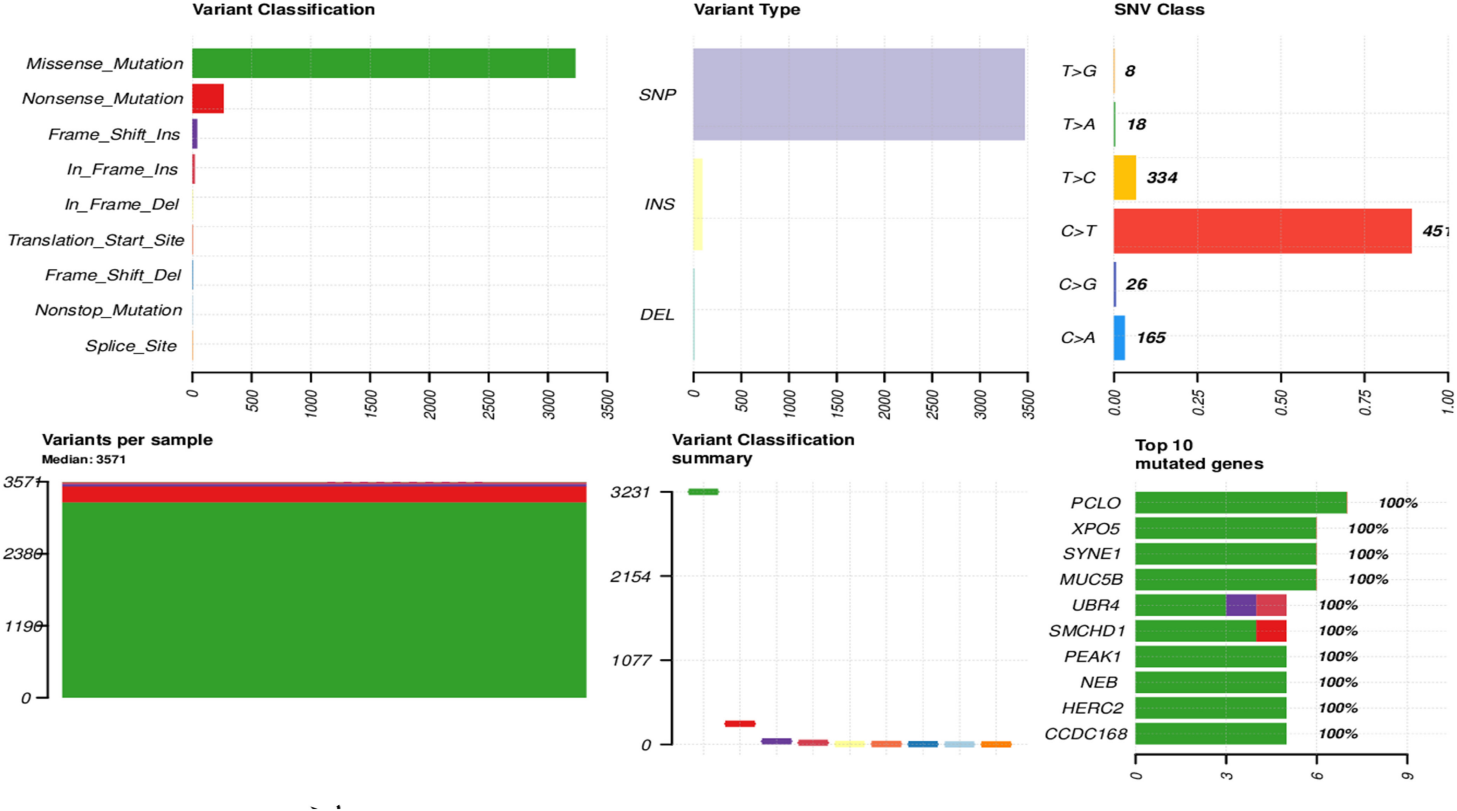
Statistical result plot of Somatic mutation types: a total of six figures are included, the upper left one represents the column chart of all samples with the functional classification of eight mutations, and the upper left figure represents the histogram of the various type in different regions of the genome. The upper left of the figure is the histogram of the mutation spectrum distribution. The lower left of the figure represents each sample's column chart of mutation function classification, the lower‐left two represent the box line diagram of eight mutation function classification, and the lower‐left third figure represents the first 10 high‐frequency mutation gene distribution histogram.

**FIGURE 6:**
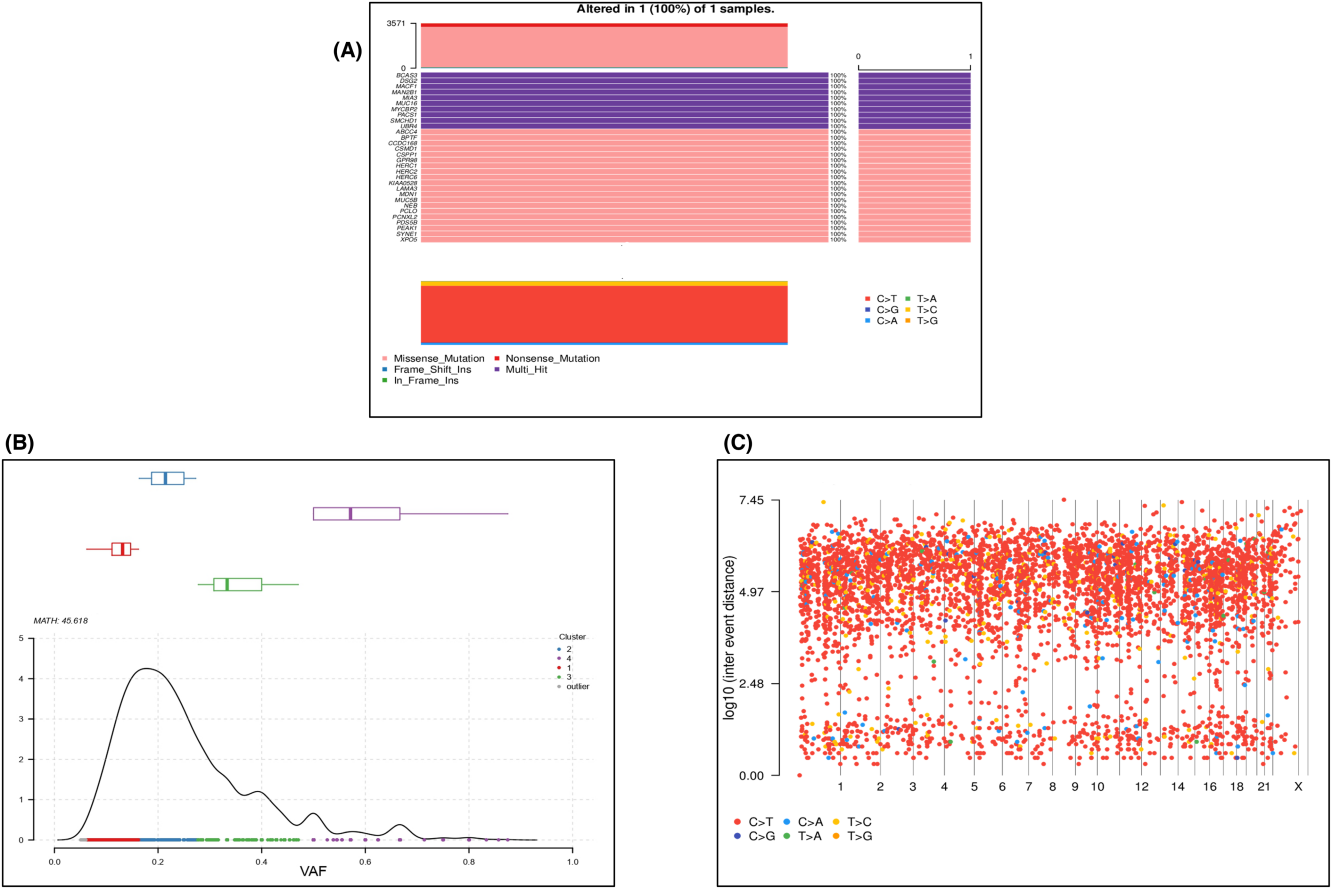
(A) Oncoplot (waterfall) illustrating the most frequently mutated genes in decreasing order of frequency of the plot also shows the type of mutation, C > G was the most common nucleotide base change. (B) Tumor heterogeneity shows the genes grouped into four group clusters and one outliner group; group 4 shows high heterogeneity, whereas it is followed by group 2 which contains more genes. (C) In the rainfall plots, each dot represents a unique somatic mutation in an individual cancer sample ordered on the horizontal axis according to its position in the human genome. The vertical line is used to calculate the genomic gap between each mutation and the mutant before.

**FIGURE 7:**
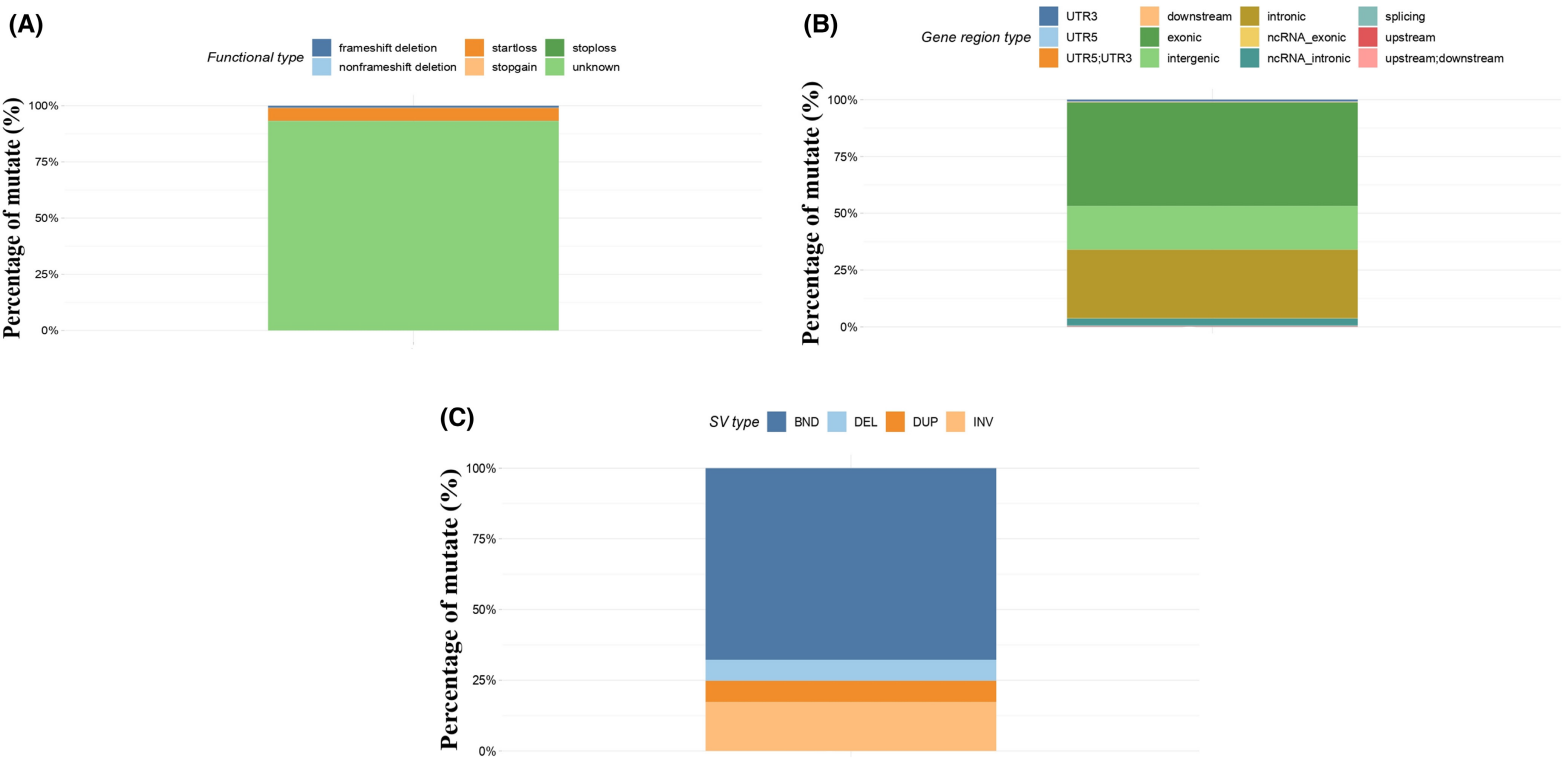
Summary of the SV variant state that we found in our study. (A) The functional type most commonly was unknown, followed by start loss (B)‐gene region type most commonly exonic then intronic, and (C) is the SV types.

After obtaining all potential polymorphic SNP/InDel mutation sites in all exons through GATK, we then further filter and screen according to factors such as quality value, depth, and repeatability and finally obtain a high‐confidence mutation data set, and then annotate it with ANNOVAR. In general, the number of alleles in the primary bladder SRCC was 5283 (5066, 102) in SNPs and indels, respectively. We found 3231 missense mutations and 264 nonsense mutations. Interestingly, we detected somatic mutations in genes that had not previously been identified with primary bladder SRCC, the following genes with more than five mutation types (PLCO, MUC5B, SYNE1, CCDC168, HERC2, NEB, PEAK1, SMCHD1, UBR4) (Figure 5). We import the obtained SNP‐InDel mutation results into the R‐tools package to perform related mutation analysis and draw oncoplot (waterfall) graphs (Figure 6A). CNVs are defined as changes in the copy number of genomic segments and are the main source of structural variation (SV), which is classified into two categories: deletion and duplication. To detect somatic CNVs, the Control‐FREEC or VarScan software was employed. We discovered 63 CNVs with gain counts and 181 with loss counts. The function type was distributed as 349 frameshift dilatation, 7 nonframeshift deletions, and 1 unknown. While the gene region type was commonly exonic with 356 vs. 1 intergenic, on the other hand, SV can be divided into insertions, deletions, inversions, and translocations (either inter‐ or intrachromosomal). It can also be complex, involving multiple events at the same location (Figure 7), we summarize the SV variant state that we found in our study.

**FIGURE 8:**
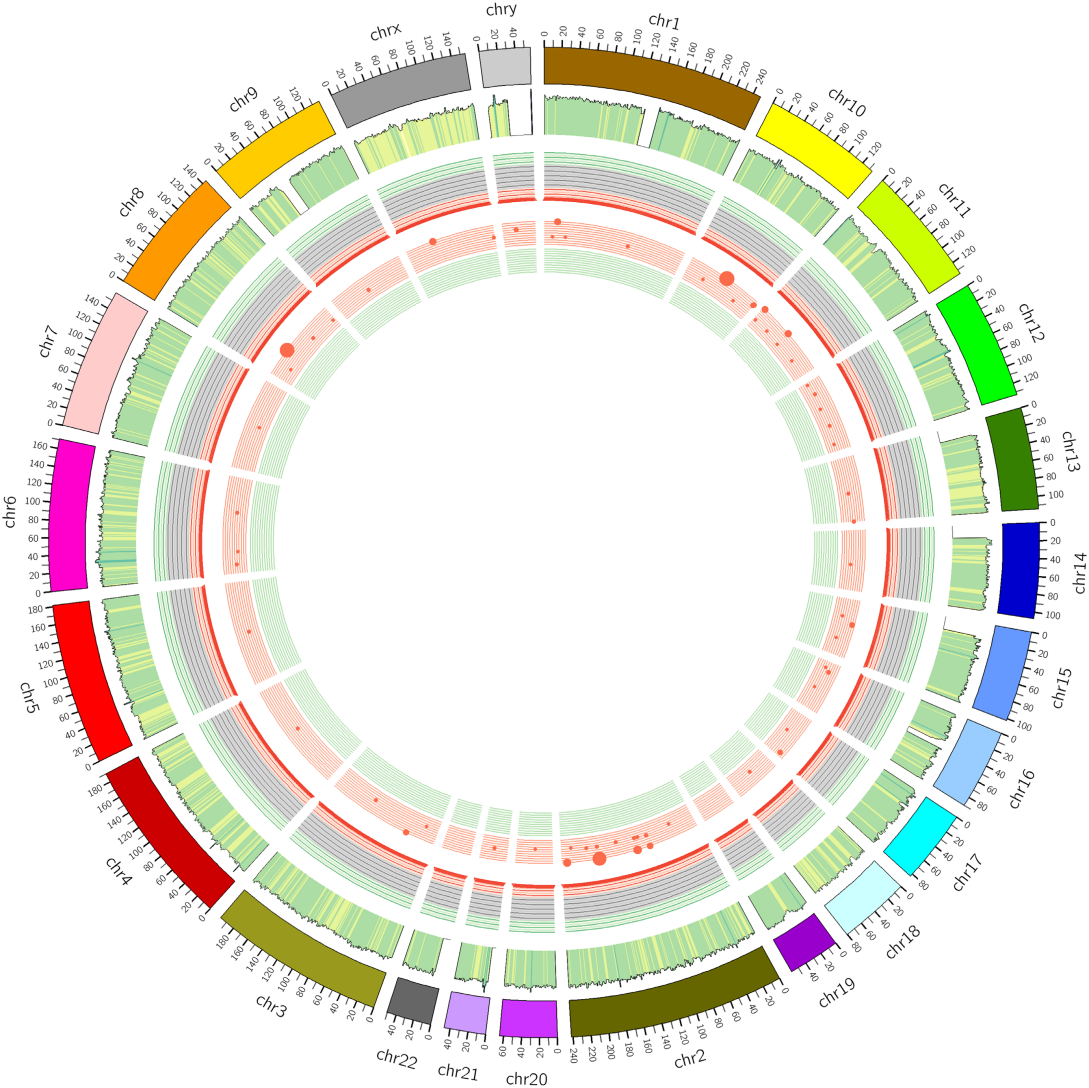
Mutation Overview Circle Diagram: The Circos diagram represents from the outermost to the innermost: The first circle is the chromosome name and corresponding karyotype; the second circle represents coverage on each chromosome; the third circle is the density of SNP‐InDel (1 Mb), the density gradually decreases from the outside to the inside, and the density is normalized; the fourth circle is the density of copy number variation (1 Mb), which is divided into two layers: the inner and outer layers, the red is the copy number increase, the green is the copy number loss, the larger the number, the larger the point; and the fifth circle is the SV between different chromosomes, and the connection line represents the phenomenon of fusion of two position genes.

The transformation/transmutation ratio (Ts/Tv) can be used to determine the correctness of the SNP data set. The ratio was approximately 0.5:2.01 in primary bladder SRCC and normal tissues. According to the principle of permutation and combination, SNP can have a a total of 6 substitution situations, namely A < ‐>T, A < ‐>G, G < ‐>T, C < ‐>T, C < ‐>G, and A < ‐>C. Due to structural reasons, the probability of conversion is higher than that of mutation. The ratio of conversion to mutation can provide information on the degree of precision with which the SNP was detected. The ratio in the whole gene is about 2.2, and the ratio in the coding region is about 3.2, and we found 4515 mutations in C > T with more than 89%. For more details about the variants, we presented the distribution of chromosome‐based variants. The Circos tool illustrated the somatic cell variation in the SRCC sample. In the histogram, the color‐coded indicate the mutations occurred in protein‐coding genes, whereas noncolored occurred in noncoding genes (Figure 8). In the histogram, the color‐coded indicate the mutations occurred in protein‐coding genes while noncolored occurred in noncoding genes.

#### 3.2.4 Enrichment analysis of known carcinogenic pathways

We collected existing carcinogenic (33 types of cancer) signaling pathways from TCGA cohorts and checked the enrichment of known carcinogenic pathways. We found that RTK‐RAS has 85 genes involved in carcinogenic signaling pathways, followed by NOTCH with 71 genes and 68 genes for WNT. The rest of the pathways are listed in Table S1.

#### 3.2.5 Kataegis‐rainful analysis

The whole cancer genome mutation catalogs were searched for clusters of mutations. Kataegis foci of localized substitution hypermutation are defined by clusters of C > T and/or C > G mutations that are significantly enriched at TpCpN trinucleotides and on the same DNA strand. Kataegis foci include between a few and several thousand mutations and are frequently discovered in close proximity to chromosomal rearrangements. Different tumors impact different genetic areas, starting with the baseline sequence of sites, adding six successive mutations, and determining the average mutation distance. Moreover, the tumor heterogeneity in primary bladder SRCC in clusters of four groups with Math = 45.618 and median absolute division = 6.837, tumor heterogeneity showed the genes grouped into four group clusters and one outliner group, the group 4 showed high heterogeneity followed by group 2 which contain more 1523 genes (Figure 6B,C).

#### 3.2.6 Analysis of predisposing genes

**FIGURE 9:**
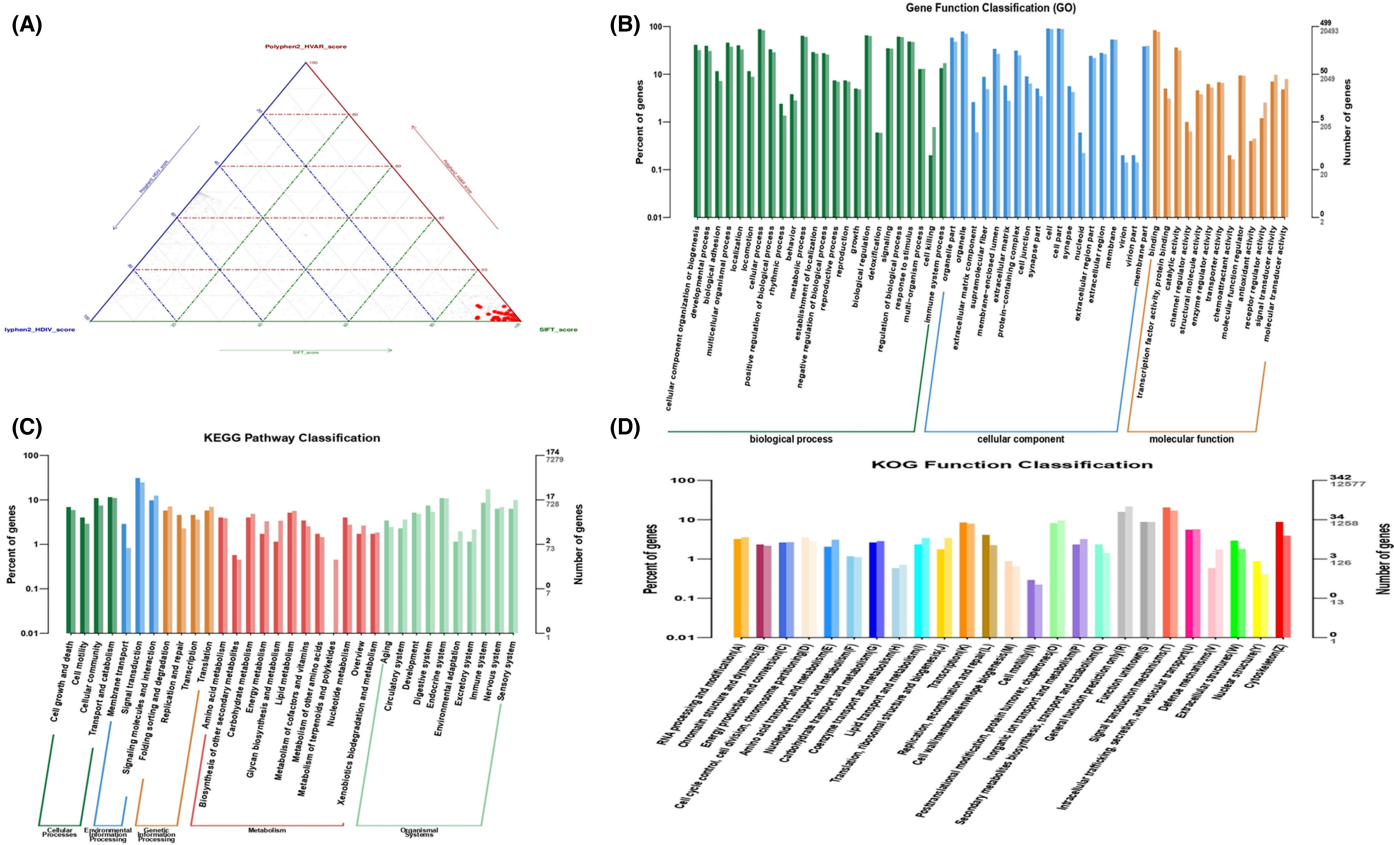
(A) The highly negative mutant prediction gene in our study was mainly screened based on the mutation classification predicted by SIFT (score < =0.05) and PolyPhen2_HVAR (score > =0.909). (B) The bar plot of predisposing genes' functional classification annotated in GO databases. The abscissa is the function classifications of the GO database and the ordinate is the number of DEGs annotated in it. The vertical axis represents the function annotation information, and the horizontal axis represents the enrichment degree corresponding to the function. (C) The bar plot functional classification annotated in KEGG significantly enriched functions. (D) The bar plot of functional classification annotated in COG and KOG databases.

According to the ACMG scoring annotation results, the annotations are reserved as harmful, potentially harmful, and ambiguous sites, we found in 1969 genes, final screening is performed on the basis of ACMG, and the mutation annotation sites are obtained by screening after retaining the harmfulness and uncertain significance score factors and the filter conditions of susceptibility genes. The SIFT score between 0.0 and 0.05 revealed 504 genes. Moreover, we adjusted the SIFT score to 0 and the PolyPhen2_HVAR to 0.986–1 to provide the most genes that are confidently predicted to be deleterious, and the results were eight genes as follows (PHEX, DMD, CCDC22, DGAT2L6, ADGRG4, SOX3, PASD1, and NAA10). The poor mutant prediction gene in our study was mainly screened based on the mutation classification predicted by SIFT (score ≤ 0.05) and PolyPhen2_HVAR (score ≥ 0.909) (Figure 9A). We found that most of the functionally enriched pathways for predisposing genes in primary bladder SRCC were extracellular matrix components, cellular component organization or biogenesis, and developmental process (Figure 9B). KEGG pathway analysis indicated that signal transduction, transport and catabolism, and cellular community were primarily enriched in the primary SRCC (Figure 9C). KOG pathway has shown that signal transduction mechanism, general function prediction, and transcription are primarily enriched (Figure 9D).

#### 3.2.7 Analysis of driver gene

**FIGURE 10:**
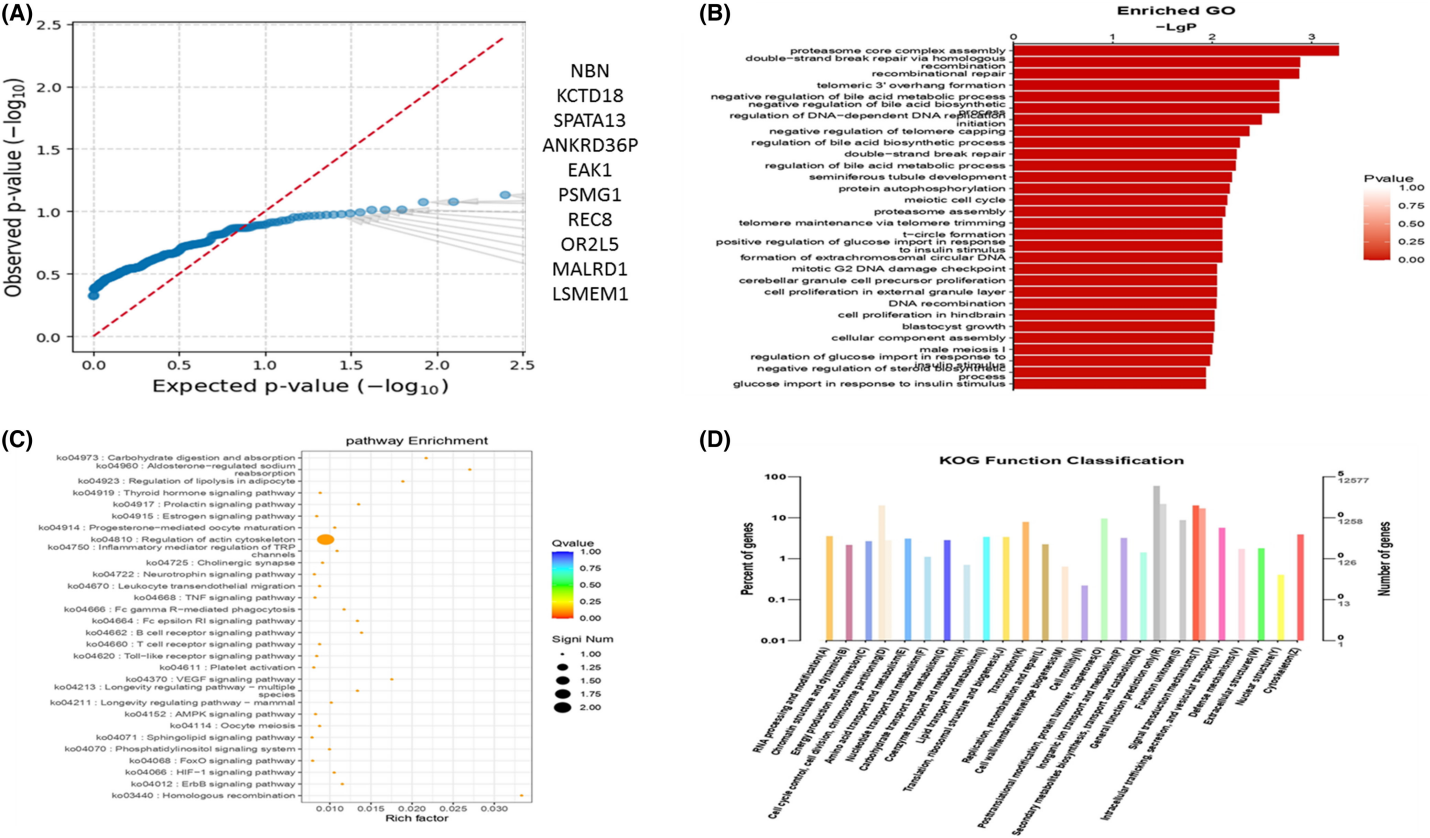
(A) Extracting the potential driver genes. The mutations of most significant10 driver genes. (B) Bar graph of a statistically significant enrichment function: the vertical line represents the data about the functional signature, whereas the horizontal line reflects the amount of enriching associated with the functional. The degree of enrichment dictates the length of the column and the depth of the color (only the top 30 GOs with the highest enrichment degree are selected for drawing).(C) Scatter plot of KEGG significantly enriched functions: the vertical axis represents the function annotation information, and the horizontal axis represents the rich factor corresponding to the function (the number of variant genes annotated to the function divided by the number of genes annotated to the function). The size of the Q value is represented by the color of the dot, and the smaller the Q value, the color the closer to red, and the number of variant genes contained in each function is represented by the size of the dots (only the top 30 GOs with the highest enrichment degree are selected for drawing). (D) The bar plot of driver genes' functional classification annotated in COG and KOG databases.

**FIGURE 11:**
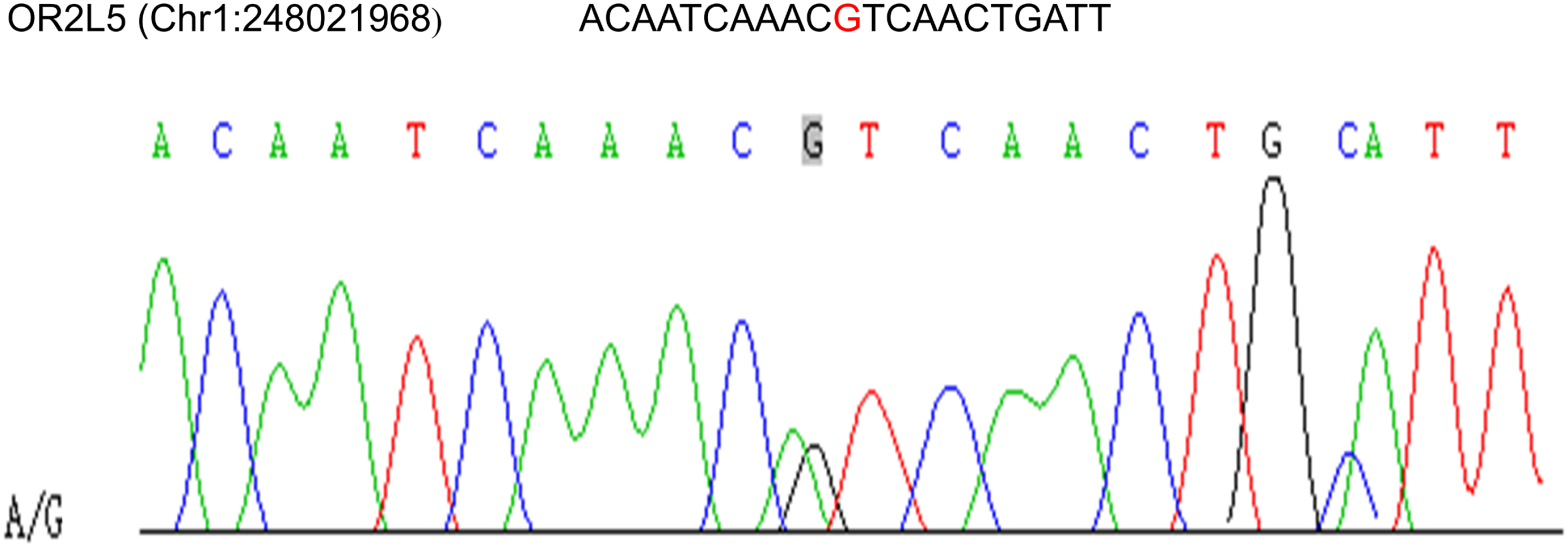
Results of nucleotide Sanger sequencing analysis. Sanger sequencing electropherograms of the OR2L5 mutant at position Chr1:248021968 A/G.

In this tumor sample, we compared somatic variance with known driver genes and filtered the known driver genes. After that, we use the SNP and InDel information obtained from the previous analysis of all samples to extract potential driver genes. Mutations in 10 driver genes are NBN, KCTD18, SPATA13, ANKRD36, PEAK1, PSMG1, REC8, OR2L5, MALRD1, and LSMEM1 (Table S2; Figure 10A). We found that most of the functionally enriched pathways for diver genes in primary bladder SRCC were cellular component organization or biogenesis (Figure 10B). KEGG pathway analysis indicated that signal transduction, immune system, and signaling molecules and interaction were primarily enriched in the primary SRCC (Figure 10C). KOG pathway has shown that general function prediction is only primarily enriched (Figure 10D). Sanger sequencing of germline DNA revealed the presence of a mutant base A/G of OR2L5 in the sequence, which was discovered for the first time in primary SRCC of the bladder (Figure 11).

After comparing the resulting driver genes to the DEGs of the ordinary transitional cell bladder cancers from TCGA, we found no similar genes between the two pathologies (Figure S2).

### 3.3 Population from SEER‐based study

#### 3.3.1 General patient's characteristics

A total of 119,689 patients, including 595 patients with SRCC and 119,094 patients with UC, were identified in the SEER database during 1975–2019, including the baseline characteristics of these patients. SRCC patients were diagnosed at a younger age at a rate of 56.6%, whereas UC patients were primarily elderly at a rate of 60.6%. The majority of patients were males in both the SRCC (71%) and the UC (74.7%) groups *P* = 0.02. The SRCC group presented with a more distant stage than the UC group (28.9 vs. 8.0%, *P* < 0.001). Higher‐grade disease was more common in the SRCC group (73.9vs. 63.3%, *P* < 0.001). The SRCC group had a lower rate of surgery than the UC group (83.4 vs. 89.4%, *P* < 0.001), with a majority of patients in both groups undergoing TURBT (32.1% vs. 48.7%, *P* < 0.001), whereas lymph nodes were more likely to be removed in the SRCC group than in the UC group (28.4 vs. 11.3%, *P* < 0.001). Moreover, when radiation sequence with surgery was known, no sequence treatment was more frequently used in both the SRCC group and the UC group (74.8 vs. 78.9%, *P* < 0.001) (Table 3).

**TABLE 3:** Demographic characteristics of bladder UC vs. SRCC groups, KM survival between the variables bladder UC vs. SRCC groups

Groups \ Variables	SRCC *n* = 595(%)	UC *n* = 119,094(%)	*P* value	Groups \ Variables	SRCC Median months	UC Median months	*P* value
Age			0.0001	Age			
25–69	337 (56.6)	46,945 (39.4)		25–69	17	96	0.0001
70‐ > 85	258 (43.4)	72,149 (60.6)		>70	11	27	0001
Race			0.0001	Race			
White	485 (81.5)	105,672 (88.7)		white	14	42	0.0001
Black	73 (12.3)	7554 (6.3)		Black	12	23	0.0001
Other	37 (6.2)	5868 (4.9)		Others	28	67	0.0001
Sex			0.02	Sex			
Female	172 (29)	30,131 (25.3)		female	13	28	0.0001
Male	423 (71)	88,963 (74.7)		Male	15	45	0.0001
Primary site			0.671	Primary site			
Trigone of bladder	33 (5.5)	7134 (6)		Trigone of bladder	15	32	0.02
Dome of bladder	57 (9.6)	5380 (4.5)		Dome of bladder	22	43	0.06
Lateral wall of the bladder	111 (18.7)	31,802 (26.7)		Lateral wall of the bladder	22	54	0.0001
Bladder neck and Ureteric orifice	23 (3.9)	7439 (6.2)		Bladder neck and Ureteric orific	17	44	0.01
Urachus	36 (6.1)	19 (0.02)		Urachus	38	29	0.86
Overlapping lesion of bladder	86 (14.5)	16,058 (13.4)		Overlapping lesion of bladder	13	21	0.017
Bladder, NOS	249 (41.8)	51,262 (43)		Bladder, NOS	11	42	0.0001
Grade			0.0001	Grade			
Grade I‐II	15 (2.5)	19,527 (16.4)		Grade I‐II	39	100	0.007
Grade III‐IV	440 (73.9)	75,459 (63.4)		Grade III‐IV	14	26	0.0001
Unknown	140 (23.5)	24,108 (20.2)		Unknown	16	63	0.0001
Seer historic stage			0.0001	Seer historic stage			
Distant	172 (28.9)	9612 (8.1)		Distant	7	5	0.201
Localized	90 (15.1)	57,264 (48.1)		Localized	36	84	0.0001
Regional	308 (51.8)	43,953 (36.9)		Regional	21	20	0.496
Unstaged	25 (4.2)	8265 (6.9)		Unstaged	12	54	0.01
Type of surgery			0.0001	Type of surgery			
No surgery	85 (14.3)	9240 (7.8)		No surgery	5	22	0.0001
TURBT	191 (32.1)	58,031 (48.7)		TURBT	11	41	0.0001
Partial cystectomy	47 (7.9)	1750 (1.5)		Partial cystectomy	43	44	0.242
Radical cystectomy	98 (16.5)	9994 (8.4)		Radical cystectomy	43	39	0.262
Pelvic exenteration	95 (16.0)	6406 (5.4)		Pelvic exenteration	22	41	0.003
Others	79 (13.3)	33,673 (28.3)		Other types of surgery	17	46	0.0001
Lymph node removal			0.0001	Lymph nodes removed			
No/unknown	426 (71.6)	105,632 (88.7)		None	13	41	0.0001
More than one	169 (28.4)	13,462 (11.3)		More than one	22	42	0.0001
Radiation sequence with surgery			0.0001	Radiation sequenced with surgery			
No sequence	445 (74.8)	94,012 (78.9)		No sequence at all	15	47	0.0001
After	77 (12.9)	10,216 (8.6)		sequence After surgery	12	16	0.009
Before and after	5 (0.8)	206 (0.2)		sequence Before and after surgery	10	32	0.001
Before	3 (0.5)	833 (0.7)		sequence Before surgery	93	38	0.995
Other	65 (10.9)	13,827 (11.6)		Others	20	42	0.019
Surgery			0.0001	Surgery			
No	99 (16.6)	12,606 (10.6)		No/unknown	5	22	0.0001
Yes	496 (83.4)	106,488 (89.4)		Yes	18	43	0.0001
Radiotherapy			0.0001	Chemotherapy			
No/unknown	489 (82.1)	106.411 (89.3)		No/unknown	15	45	0.0001
Yes	106 (17.8)	12,685 (10.6)		Yes	14	27	0.0001
Chemotherapy			0.0001	Others	19	23	0.018
No/unknown	384 (64.5)	97.962 (82.2)		Radiotherapy			
Yes	211 (35.5)	21,132 (17.8)		No/unknown	16	49	0.0001
Specific cause of death			0.0001	Yes	11	15	0.0001
Cancer‐specific death	291 (48.9)	33,891 (28.5)		Others	20	34	0.03
Other causes of death	304 (51.1)	85,203 (71.5)		Death Cause specific			
Insurance			0.119	Same cancer cause	10	12	0.0001
Insured	205 (34.5)	38,239 (32.1)		Other cause of death	31	83	0.0001
Others	390 (65.5)	80,857 (67.9)		Insurance			
Marital status				No/unknown	15	29	0.0001
Married	317 (53.3)	62,583 (52.5)	0.001	Insured	11	38	0.0001
Not married	213 (35.8)	37,056 (31.1)		Others	23	42	0.33
Others	65 (10.9)	19,457 (16.3)		Marital status			
				Married	15	55	0.0001
				Others	20	48	0.016

#### 3.3.2 Survival analysis

**FIGURE 12:**
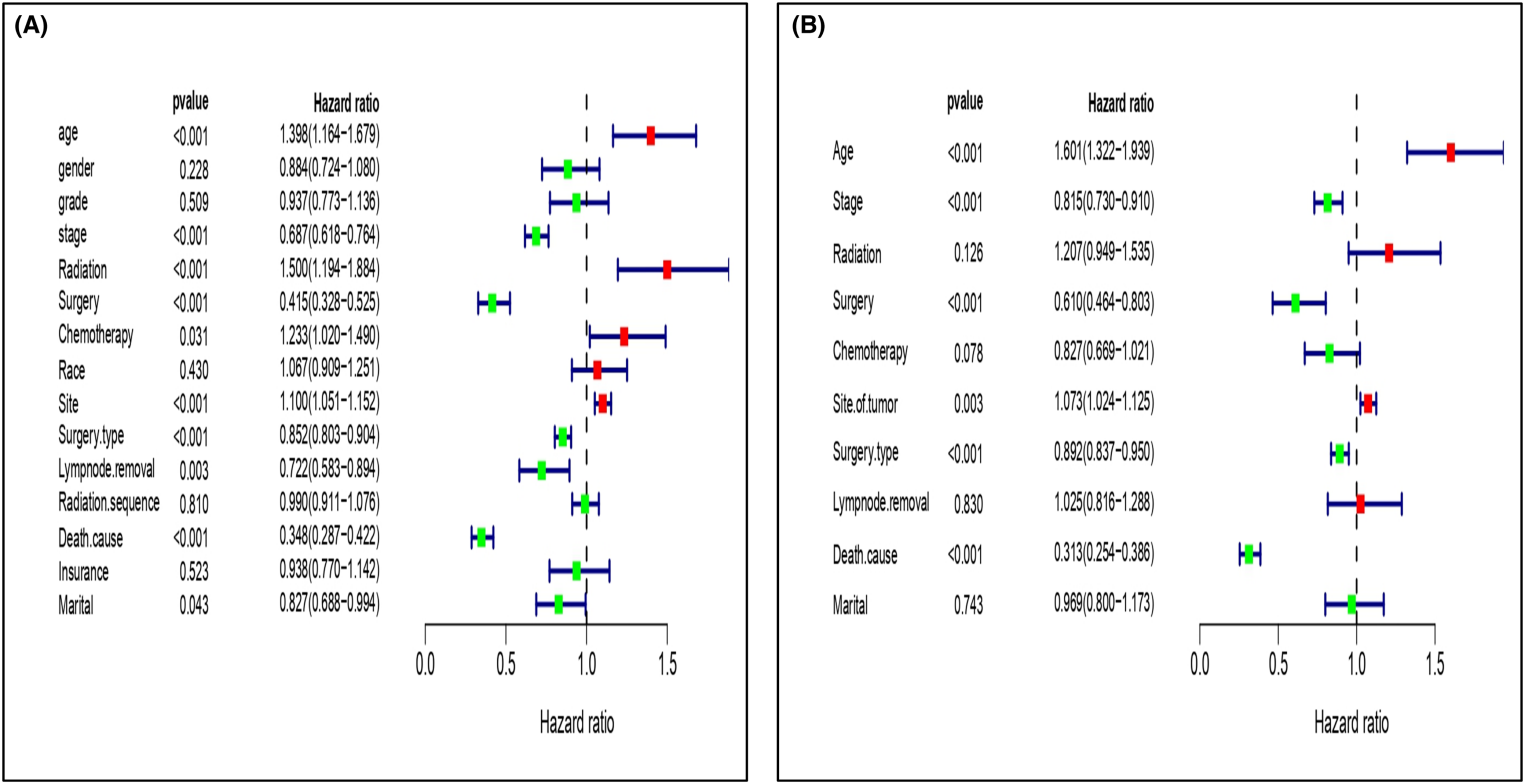
(A) In the univariate study of SRCC, all variables such as age, race, sex, primary site, grade, stage, type of surgery, lymph node removal, radiation sequenced with surgery, surgery performed, radiotherapy record, chemotherapy record, insurance, and marital status were included. (B) The variables with significant value were subsequently added to the multivariate Cox analysis which was a risk factor for survival.

By using KM, the SRCC exhibited lower OS than the UC (14 median months vs. 41 median months; *P* = 0001), even after adjusting the variables in the Cox regression, the SRCC of bladder showed worse survival HR = 1.119, 95% CI = 1.081–1.328, *P* = 0.0001. Furthermore, there was a statistically significant difference in survival between the variables with better survival, except for primary site (urachus), type of surgery (radical cystectomy), and radiation sequenced with surgery (sequence radiation before surgery) that had survival benefits for SRCC patients, all variables showed better survival in UC patients (Table 3). Univariate analysis of SRCC and UC, age, race, sex, primary site, grade, stage, type of surgery, lymph node removal, radiation sequenced with surgery, surgery performance, radiotherapy status, chemotherapy status, a specific cause of death, insurance, and marital status were risk factors for survival (*P* < 0.001), the significant values were included in the multivariate Cox analysis (Figure 12).

## 4 DISCUSSION

SRCC is related to distinct epidemiology and oncogenesis in gastric and colon cancer; however, no comparable study on anticipated genetic variables has been undertaken in bladder cancer.^12^ Primary SRCC most frequently occurs in the gastric 63.4%, the colon 18.2%, the lung 3.1%, the pancreas 1.8%, the breast 1.5%, and the bladder 1.3%.^13^The most current World Health Organization categorization divides bladder cancer into microscopically different subgroups. Moreover, the genesis and course of ordinary bladder urothelial carcinoma are primarily driven by DNA‐level molecular alterations. Several genome‐wide studies have recently sought to analyze DNA changes associated with urothelial carcinoma focusing on those originating in the bladder.

The most frequently mutated genes in ordinary bladder cancer are TP53x, KMT2A, SPTAN1, ERBB2, CREBBP, FAT1, ATM, and KMT2C. Additionally, 158 genes were identified as epigenetically suppressed using DNA methylation and gene expression analysis, including SPATC1L (19%), nicotinate phosphoribosyltransferase (NAPRT) (13%), poly (ADP‐ribose) polymerase PARP6 (26%), and latexin (LXN) (27%).^14^, ^15^, ^16^, ^17^ On the other hand, primary SRCC of the bladder is described in the literature as case reports or brief series, with no examination of molecular features or mean driver genes.^18^ Our case study was confirmed by a pathologist from our hospital by immunohisochemstry and indicated the expression of CK7, CK20, and GATA‐3 and the expression of CK (AE1/AE2), EMA, and Ki67 were observed in primary SRCC of the bladder.

Whole‐genome sequencing is competitively priced, offers timely service, and offers comprehensive genomic coverage. The SNP‐InDel filtering results for each sample are shown, with the majority of annotations based on genes, genomic areas, and functions corrected for gene size, expression level, sample‐specific mutation rate, and nonsynonymous to synonymous mutation ratio. Even if a mutation does not meet the statistical significance criteria, it may be functionally important. However, based on the frequency of mutations significantly detected throughout our analysis, nine genetic variants have been identified as being highly mutated in primary bladder SRCC (PLCO, MUC5B, SYNE1, CCDC168, HERC2, NEB, PEAK1, SMCHD1, UBR4). In analyzing certain molecular signatures in different organs, the molecular genesis of SRCC is of considerable interest. For example, in colorectal SRCC, loss of E‐cadherin protein expression and a higher frequency of KRAS were noticed compared to conventional adenocarcinoma in the same study. In addition, the gastric SRCC E‐cadherin (CDHI) gene was prominent.^19^, ^20^, ^21^ Reduced E‐cadherin expression due to aberrant hypermethylation is important for cancer metastasis. In our study, a mutation on CDHI at chromosome 16 was identified.^22^ Park and his colleagues exposed that SOX2 and MUC2 are mucin‐related genes involved with the etiology of colorectal SRCC, and we confirmed this finding in our primary SRCC mutant genes, in chromosome 3 for MUC1, chromosome 9 for SOX2, and MUC5B, which has 6 mutations.^23^, ^24^, ^25^ Driver genes, on the other hand, play a role in the start and course of the disease. Patients can be identified and targeted for adapted precision medical therapy after discovering the driving genes.^26^ Our analysis excluded genes with mild or benign anticipated functional effects using (SIFT) and Polyphen2 scores, which revealed the most 10 accurate and significant genes with carcinogenic influence (NBN, KCTD18, SPATA13, ANKRD36, OR2L5, MALRD1, and LSMEM1). The analysis of the mean pathway revealed that RTK‐RAS, NOTCH, and WNT, which involved carcinogenic signaling pathways, were prevalent in primary SRCC, where previously mentioned to have a role and present in colorectal SRCC, urothelia, and gastric SRCC.^27^, ^28^, ^29^, ^30^, ^31^ However, some report human bladder cancer samples exhibit a very low incidence of H‐Ras mutations in the formation of urothelial cancer.^32^

Furthermore, our reported driver genes were validated by Sanger sequencing, which resulted in the finding of OR2L5 for the first time in primary SRCC of the bladder. This finding should open the way for more studies about this tumor and related treatments. The human olfactory receptor (OR) gene family has functions in several physiological processes including cancer. In myelogenous leukemia, OR2AT4 promotes cell proliferation, apoptosis, and differentiation. OR2T6 is overexpressed in breast cancer tissues, whereas OR51E2 is overexpressed in prostate cancer and enhances tumor cell invasiveness via PTEN loss.^33^, ^34^, ^35^, ^36^ By comparison, we analyzed the DEG of the ordinary transitional cell bladder tumors from TCGA, then compared them with the most 10 significant driver genes of SRCC and revealed that no equivalent gene exists between the two pathologies. However, by classifying the driver genes with low scores, we can uncover numerous driver genes, such as TP53, FGFR3, ARID1A, KDM6A, CDKN2A, and others, that have a mild or benign influence on SRCC. Cazier et al., on the other hand, demonstrated that these genes are extremely carcinogenic in ordinary bladder cancer.^27^ Finally, by using the last updated edition of the SEER database, the survival comparison between SRCC and UC revealed that ordinary urothelial carcinoma has a greater survival rate, which will explain the difference in gnomic characteristics and driving pathways between the two diseases.^37^, ^38^, ^39^

But there are limitations to our analysis. First, due to the rarity of the disease, only a small number of cases were available for analysis. A multicenter study is required to explore the clinical characteristics of primary SRCC in the urinary bladder. Second, more global analyses, particularly RNA‐seq, would bolster the content of the study. Furthermore, while WES was utilized to analyze probable driver mutations, further investigation into the particular mechanism of people with different or functional data is necessary, which will be accomplished using primary cell culture technologies obtained from primary SRCC tissues in the bladder. This might be advantageous in the evaluation of genetic changes. However, as a result of the restricted number of samples available for WES, it is difficult to totally prevent false discovery rates associated with the impact of numerous tests. As a result, our hypotheses must be extended or clarified in greater detail.

## 5 CONCLUSION

To the author’s knowledge, this is the first study that investigates the molecular biology of the primary signet ring cell of the urinary bladder; as a result of genomic advances, the whole‐genome sequence has evolved to become the cutting‐edge method for finding novel cancer drivers and predisposing genes. In our study, we found several driver genes including NBN, KCTD18, SPATA13, ANKRD36, OR2L5, MALRD1, and LSMEM1. Sanger sequencing of germline DNA revealed the presence of a mutant base A/G of OR2L5 in the sequence, which was discovered for the first time in primary SRCC of the bladder. Moreover, we found eight highly predicting predisposing genes (PHEX, DMD, CCDC22, DGAT2L6, ADGRG4, SOX3, PASD1, and NAA10). RTK‐RAS, NOTCH, and WNT were the significant pathways that enriched more mutated genes.

## AUTHOR CONTRIBUTIONS

Mohammed Alradhi, Mohammed Safi, and Abdullah Al‐danakh contributed to the study concept and design, undertook project leadership and guaranteed this work. Mohammed Alradhi, Bo Fan, and Xiancheng Li, were responsible for data collection and interpretation. Mohammed Alradhi, Mohammed Safi, controlled quality of data and algorithms. Mohammed Alradhi, Mohammed Safi, Abdullah Al‐danakh, analyzed data and interpreted the data. Mohammed Alradhi, Shuang Wen wrote the first Xiancheng Li, Min Sun. Shuang Wen, and Mohammed Alradhi reviewed and the manuscript. The writing revised draft: Shuang Wen, Hong long Wang, Mohammed Safi, Abdullah Shopit. All authors contributed to the article and approved the submitted version.

## FUNDING INFORMATION

The present research was supported by grants from the National Natural Science Foundation of China (grant nos. 81972831 and 31800787), the Dalian High‐level Talents Innovation Support Program (grant no. 2019RQ014), the United Fund of the Second Hospital of Dalian Medical University and Dalian Institute of Chemical Physics, Chinese Academy of Sciences (grant no. UF‐QN‐202004), and The Young Reserve Talent Project of the Second Affiliated Hospital of Dalian Medical University (dy2yhbrc202010).

## CONFLICT OF INTEREST

The authors declare that they have no conflict of interest.

## ETHICS STATEMENT

The study protocol was approved by the Ethics Committee of the Second Hospital of Dalian Medical University and followed the Declaration of Helsinki Ethical Principles for Medical Research Involving Human Subjects, in accordance with Dalian's ethics committee for human research and the patient's written consent was taken.

## Supporting information


Figures S1‐S2

Table S1‐S2

## Data Availability

TCGA data was uploaded from http://gepia.cancer‐pku.cn/, https://portal.gdc.cancer.gov/, and http://xena.ucsc.edu/. SEER data was uploaded from https://seer.cancer.gov/.
