# Supplementary material for: Molecular genetic and clinical characteristic analysis of primary signet ring cell carcinoma of urinary bladder identified by a novel OR2L5 mutation
Source: Cancer Med. 2022 Aug 11;12(4):3931–51. doi: 10.1002/cam4.5121 (PMC9972163; doi:10.1002/cam4.5121)

**Supplementary figure 1**

The resected bladder harboring the lesion was removed via a robotic aided radical cystectomy.**B**- Tumor slices at the 100*200*400(B,C,D) that have been stained with hematoxylin and eosin.**C**- Using the periodic acid–Schiff stain, the cytoplasmic vacuoles of the tumor cells were found to be intensely pink in color. **D**-the stained cells E,F,G. Tumor cells are infiltrating muscularis propria (H&E, 20X);Tumor cells have metastasized to the lymph node (H&E, 20X). with more than 50% of them containing mucin and pushing the nucleus to its lateral and giving the ring shape


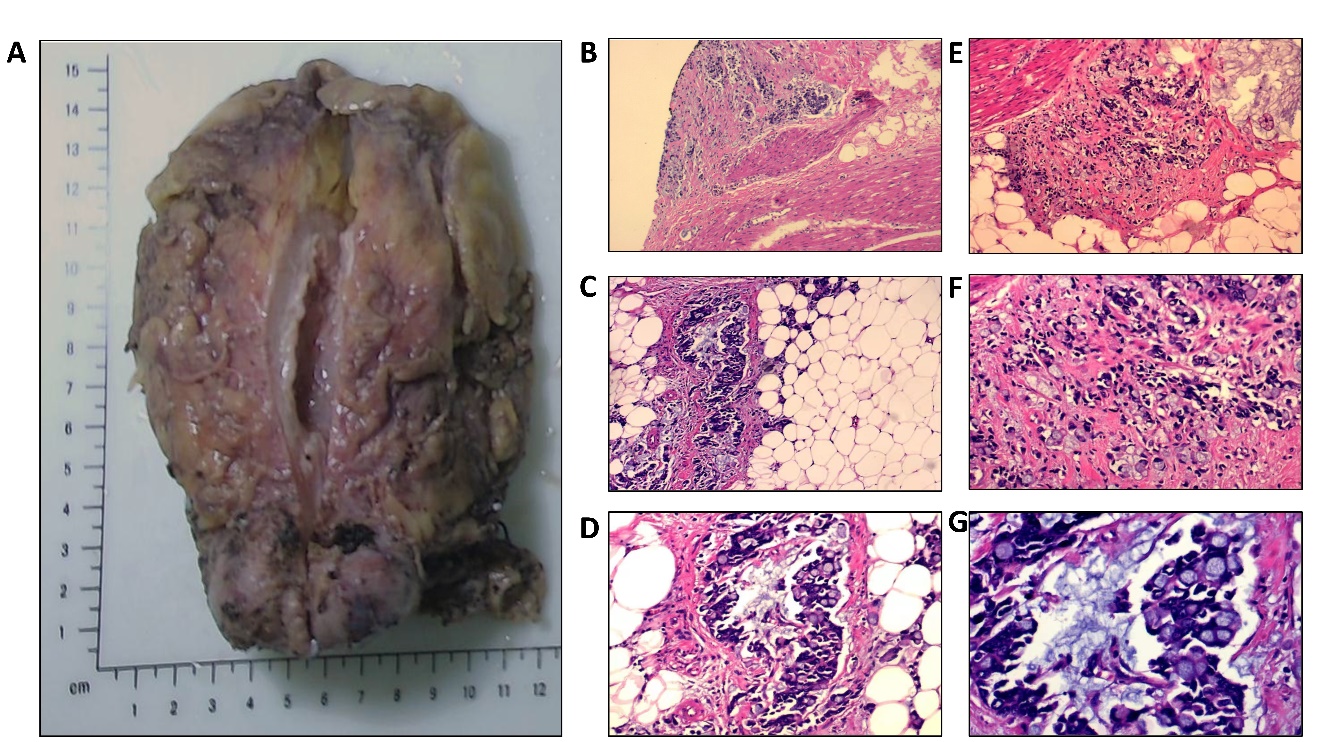


**Supplementary figure 2**

The differentially expressed genes of the ordinary transitional cell cancer of the bladder from TCGA


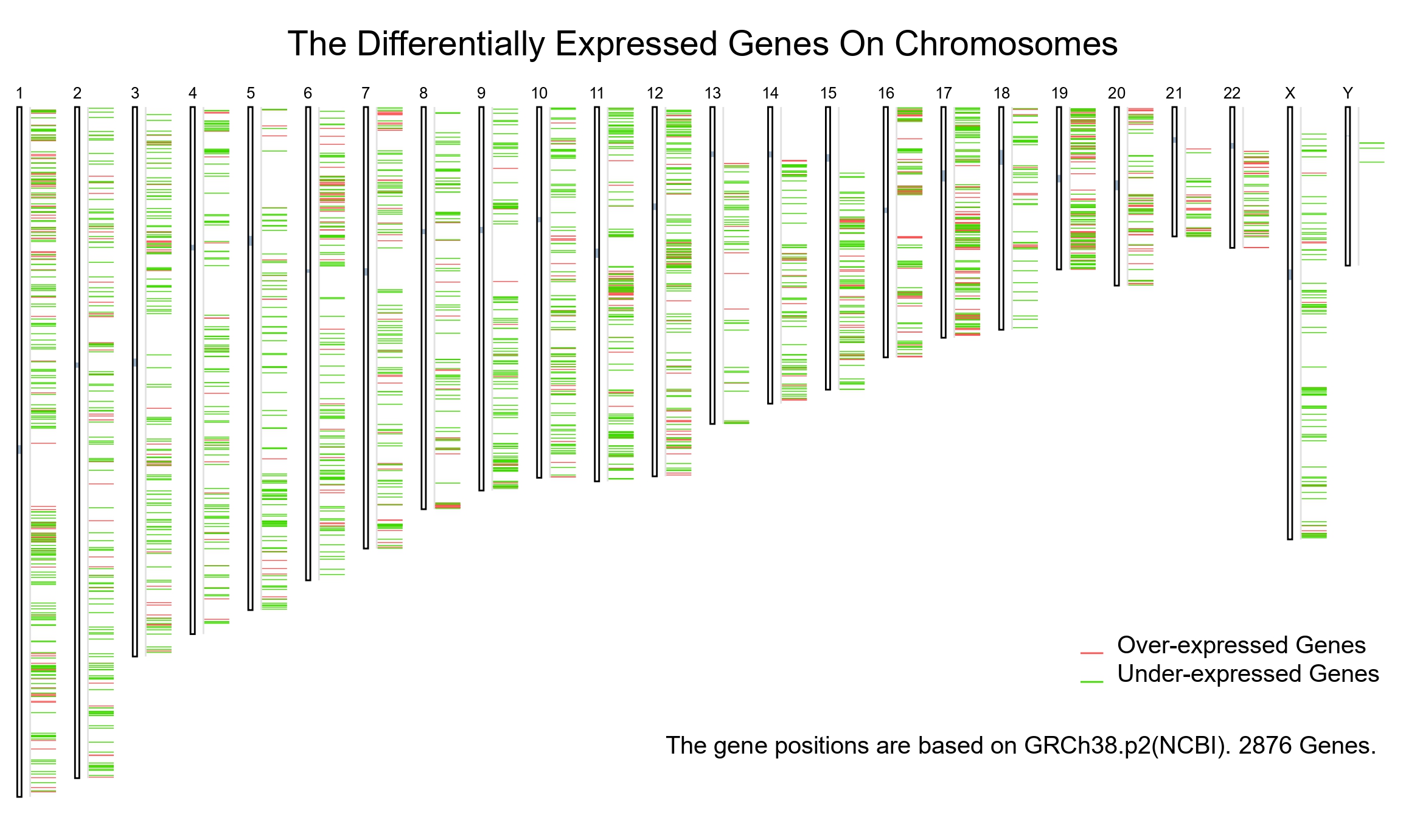

Supplement: Supplementary file 1 — Figures S1‐S2 [file CAM4-12-3931-s001.doc]
