# Supplementary material for: Molecular genetic and clinical characteristic analysis of primary signet ring cell carcinoma of urinary bladder identified by a novel OR2L5 mutation
Source: Cancer Med. 2022 Aug 11;12(4):3931–51. doi: 10.1002/cam4.5121 (PMC9972163; doi:10.1002/cam4.5121)
Supplement: Supplementary file 2 — Table S1‐S2 [file CAM4-12-3931-s002.docx]

**Supplementary table 1. Cancer pathway enrichment table**

**Pathway: TCGA cohorts collects existing carcinogenic signaling pathways, N: The number of genes involved in carcinogenic signaling pathways, n_affected_genes: The number of genes mapped, fraction_affected: The ratio of map to the number of genes**

**Supplementary table 2. Onco-drive CLUSTL mutation gene list**

| *SYMBOL* | *ENSID* | *CHROMOSOME* | *STRAND* | *LENGTH* | *TOTAL_MUTATION* | *SCORE* | *P_ANALYTICAL* |
| --- | --- | --- | --- | --- | --- | --- | --- |
| ***NBN*** | ENSG00000104320 | 8 | - | 2281 | 3 | 113.8071 | 0.074 |
| ***KCTD18*** | ENSG00000155729 | 2 | - | 1287 | 2 | 170.7107 | 0.084 |
| ***SPATA13*** | ENSG00000182957 | 13 | + | 4212 | 2 | 170.7107 | 0.0845 |
| ***ANKRD36*** | ENSG00000135976 | 2 | + | 5907 | 2 | 170.7107 | 0.097 |
| ***PEAK1*** | ENSG00000173517 | 15 | - | 5245 | 5 | 90.71068 | 0.097456 |
| ***PSMG1*** | ENSG00000183527 | 21 | - | 874 | 2 | 170.7107 | 0.0975 |
| ***REC8*** | ENSG00000100918 | 14 | + | 1663 | 2 | 141.4214 | 0.102015 |
| ***OR2L5*** | ENSG00000197454 | 1 | + | 940 | 3 | 113.8071 | 0.104 |
| ***MALRD1*** | ENSG00000204740 | 10 | + | 6511 | 2 | 170.7107 | 0.1055 |
| ***LSMEM1*** | ENSG00000181016 | 7 | + | 399 | 2 | 141.4214 | 0.106528 |

STRAND: Strand of the GE ("+" or "-"), LENGTH: length (bp) of the GE, TOTAL_MUT: total substitutions observed in the GE
